# Drug-Target Interaction Prediction Based on Adversarial Bayesian Personalized Ranking

**DOI:** 10.1155/2021/6690154

**Published:** 2021-02-10

**Authors:** Yihua Ye, Yuqi Wen, Zhongnan Zhang, Song He, Xiaochen Bo

**Affiliations:** ^1^School of Informatics, Xiamen University, Xiamen 361005, China; ^2^Department of Biotechnology, Beijing Institute of Radiation Medicine, Beijing 100850, China

## Abstract

The prediction of drug-target interaction (DTI) is a key step in drug repositioning. In recent years, many studies have tried to use matrix factorization to predict DTI, but they only use known DTIs and ignore the features of drug and target expression profiles, resulting in limited prediction performance. In this study, we propose a new DTI prediction model named AdvB-DTI. Within this model, the features of drug and target expression profiles are associated with Adversarial Bayesian Personalized Ranking through matrix factorization. Firstly, according to the known drug-target relationships, a set of ternary partial order relationships is generated. Next, these partial order relationships are used to train the latent factor matrix of drugs and targets using the Adversarial Bayesian Personalized Ranking method, and the matrix factorization is improved by the features of drug and target expression profiles. Finally, the scores of drug-target pairs are achieved by the inner product of latent factors, and the DTI prediction is performed based on the score ranking. The proposed model effectively takes advantage of the idea of learning to rank to overcome the problem of data sparsity, and perturbation factors are introduced to make the model more robust. Experimental results show that our model could achieve a better DTI prediction performance.

## 1. Introduction

Drug repositioning is to discover new indications for existing drugs, which means that drug development based on approved drugs does not need to consider the safety and effectiveness of the original drug, effectively reducing the time of drug development process and cost. Prediction of drug-target interaction (DTI) which refers to the recognition of interactions between chemical compounds and the protein targets in the human body has become a key step in drug repositioning [1].

Due to the high cost of conducting animal experiments and clinical trials for a new drug [2], a large number of machine learning-based methods have been widely used in DTI prediction in recent years, and the cost of drug development has been greatly reduced through rapid screening of potential drug-target combinations [3, 4].

Existing machine learning-based methods often use the features of drugs and targets for prediction [5, 6]. They treat the prediction problem as a binary classification problem [7]. Drug-target pairs with interaction are considered positive samples, while pairs without interaction are treated as negative samples. The output of the binary classification is the label with higher prediction probability [8–10]. Bleakley and Yamanishi used a support vector machine (SVM) framework based on bipartite local models (BLM) to predict DTIs [11]. Mei et al. improved the original DTI prediction framework by integrate neighbor-based interaction-profile inferring (NII) into the existing BLM method [12]. Buza and Peška extended the BLM method to predict DTIs by using the hubness-aware regression technique [13]. Laarhoven et al. proposed a Gaussian interaction profiling (GIP) kernel to represent the interactions between drugs and targets [14] and then integrated the weighted nearest neighbor method into it to predict DTIs [15]. Chen et al. proposed a Random Walk with Restart-based method on the heterogeneous network to infer potential DTI [16]. Some studies constructed a heterogeneous network which integrates diverse drug-related information to predicted DTI [17, 18]. Thafar et al. utilized graph embedding for DTI prediction [19]. Zhao et al. integrated graph convolutional network and Deep Neural Network to predict DTI [20]. Since the number of positive samples is small, the machine learning-based methods can easily learn to predict unknown samples as negative to reduce the training penalty [3]. Recommendation system is aimed at obtaining accurate prediction results of unknown data even with a small amount of observed data. Considering the problem of data sparseness, learning to rank (LTR) in the recommendation system is able to accurately predict even with a small amount of known data. Therefore, in this study, we defined the DTI prediction problem as a ranking problem. The following paragraph introduces how we define the DTI prediction problem as a ranking problem.

LTR implies a scoring mechanism in which interacting drug-target pairs should have a higher score than those without interaction. In this way, samples with higher scores are treated as interacting drug-target pairs [21, 22]. Recently, there are some studies that apply the idea of LTR to predict DTI [23, 24]. Bagherian et al. showed that matrix factorization algorithms have outperformed other methods in DTI prediction [25]. Thus, we utilized matrix factorization of LTR to predict DTI in this study. Bayesian Personalized Ranking (BPR) which is a matrix factorization of LTR approach has been shown to be an excellent approach for various preference learning tasks even when data are sparse [26, 27].

However, the existing methods do not effectively combine the features of drug and target with the matrix factorization method. Thus, in this study, we propose a DTI prediction model in which BPR is the core and combined gene expression to improve the prediction performance. In the proposed model, the principle of ordering is that interacting drug-target pairs (i.e., positive samples) should be ranked before noninteracting drug-target pairs (i.e., negative samples). Firstly, a set of ternary partial orders is generated based on the positive samples and the negative samples. The set is divided into a training set and a test set. Next, the Adversarial Bayesian Personalized Ranking (ABPR) method is used to train the latent factors of drugs and targets, and the drug-drug similarity and target-target similarity are calculated based on their features, respectively, to improve the training of the latent factors. Finally, for each drug, the inner product of drug's latent factor and target's latent factor is used as the score for ranking. The top-ranked drug-target pairs are predicted with interaction, and the bottom-ranked drug-target pairs are predicted without interaction. This study has the following three contributions:

- Aiming at the existing problem of DTI prediction, the idea of matrix factorization of LTR is introduced to process a sparse matrix
- BPR is not robust and vulnerable to adversarial perturbations on its parameters [28]. Perturbation factors are introduced to make the model more robust
- This study also uses the drug and target expression profiles to calculate the drug-drug and target-target similarity, respectively, to improve the training of latent factors

Experimental results show that our method is significantly better than the traditional DTI prediction methods, such as Deep Neural Network (DNN) [8, 29], Generalized Matrix Factorization (GMF) [30], and other state-of-the-art LTR methods, like Neural Matrix Factorization (NeuMF) [30] and Adversarial Matrix Factorization (AMF) [28].

## 2. Data and Definition

### 2.1. Data Source

The Library of Integrated Network-Based Cellular Signatures (LINCS) project is a mutual fund project administered by the National Institutes of Health (NIH). This project uses L1000 technology to generate approximately one million gene expression profiles [31]. The L1000 technology uses the correlation between gene expressions to drastically reduce the amount of gene expression that needs to be measured, from more than 20,000 to 978. In this study, we use the drug perturbation and gene knockout transcriptome data from seven cell lines including A375, A549, HA1E, HCC515, HEPG2, PC3, and VCAP. There are three reasons to choose drug perturbation and gene knockout transcriptome data as feature data of drugs and targets: (1) both drug perturbation and gene knockout transcriptome data are from LINCS project and are processed by using L1000 technology. So they are naturally suited to be combined as the feature data. (2) There is a correlation between drug perturbation transcriptome data and the drug's target gene knockout transcriptome data. Pabon et al. have verified in their work that drug perturbation-induced mRNA expression profile correlates with the knockout-induced mRNA expression profile of the drug's target gene and/or genes on the same pathway(s) [32]. The correlation reveals drug-target interactions. Therefore, the correlation based on the expression profile suggests that we can treat the expression profiles as feature data for dual similarity regularization. (3) Transcriptome data can capture the complexity of drug activity in cells. So the use of information obtained from transcriptional profiling studies has a huge impact on multiple areas of the drug discovery including target identification, validation, compound selection, pharmacogenomics, biomarker development, clinical trial evaluation, and toxicology [33].

DrugBank is a comprehensive, freely available web resource containing detailed drug, drug-target, drug action, and drug interaction information about FDA-approved drugs as well as experimental drugs going through the FDA approval process [34]. To obtain complete DTI data, PubChem ID is used as the identifier of drug in the DrugBank and LINCS databases.

The data volume for the seven cell lines is listed in Table 1. The positive drug-target interactions from DrugBank are used to generate interacting drug-target pairs. To avoid treating unknown drug-target interactions in DrugBank as negative interactions, we constructed the nontarget set that any member of this set has no interaction record with any drug from the same cell line in DrugBank. That means the pair of a nontarget and a drug from the same cell line could be more likely to be treated as a negative sample.

### 2.2. Problem Definition

In this study, DTI prediction is defined as a ranking problem of drug-target scores.

**Definition 1 .**

*D* ^*α*^ = {*d*_1_^*α*^, *d*_2_^*α*^, *d*_3_^*α*^, ⋯, *d*_*m*_^*α*^} represents the set of *m* drugs in cell line *α*, where *d*_*i*_^*α*^ = {*d*_*i*,1_^*α*^, *d*_*i*,2_^*α*^, ⋯, *d*_*i*,978_^*α*^} represents the expression profile of *i*-th drug.

**Definition 2 .**

*T* ^*α*^ = {*t*_1_^*α*^, *t*_2_^*α*^, *t*_3_^*α*^, ⋯, *t*_*n*_^*α*^} represents the set of *n* targets and nontargets in cell line *α*, where *t*_*j*_^*α*^ = {*t*_*j*,1_^*α*^, *t*_*j*,2_^*α*^, ⋯, *t*_*j*,978_^*α*^} represents the expression profile of *j*-th target or nontarget.

**Definition 3 .**

*Y* ^*α*^ represents the interaction relationship, and *y*_*i*,*j*_^*α*^ ∈ {0, 1}. If *y*_*i*,*j*_^*α*^ = 1, the pair of the drug *d*_*i*_^*α*^ and target *t*_*j*_^*α*^ is a positive sample; otherwise, *y*_*i*,*j*_^*α*^ = 0, and the pair of *d*_*i*_^*α*^ and *t*_*j*_^*α*^ is a negative sample.

As shown in Table 1, the numbers of drugs, targets, and interacting drug-target pairs in this study are all limited (for each cell line). Therefore, *Y*^*α*^ is a small-sized sparse matrix.

All combinations of drug and target with interactions in each cell line are used as positive samples; all drug and nontarget combinations are used to construct a negative sample candidate set. Since the number of negative samples is much larger than the number of positive samples in each cell line, we randomly sampled some negative samples from the negative sample candidate set to ensure that the number of selected negative samples is consistent with the number of positive samples within the same cell line.

Based on the known relationships of drug-target pairs, the score of drug-target pairs is sorted. The drug-target pairs with higher scores are more likely to interact. Conversely, the drug-target pairs with lower scores are more likely not to interact. Therefore, we transformed the DTI prediction problem into a problem that finds out a reasonable ranking strategy for a drug-target pair. In this paper, the methods are discussed in the same cell line, so the superscript *α* is omitted.

## 3. Methods

The proposed method (AdvB-DTI) is based on the method of BPR. Firstly, according to the interaction relationship *Y*, a ternary partial order set is generated as *H* = {*H*_*i*_ | 1 ≤ *i* ≤ *m*}, where *H*_*i*_ = {(*d*_*i*_, *t*_*j*_, *t*_*k*_) | *d*_*i*_ ∈ *D*, *t*_*j*_ ∈ *T*, *t*_*k*_ ∈ *T*, *y*_*i*,*j*_ ∈ *Y*, *y*_*i*,*k*_ ∈ *Y*, *y*_*i*,*j*_ = 1, *y*_*i*,*k*_ = 0}. *H*_*i*_ combines the target *t*_*j*_ of one positive sample and the target *t*_*k*_ of the corresponding negative sample with the same drug *d*_*i*_ into a partially ordered triple (*d*_*i*_, *t*_*j*_, *t*_*k*_), which means that (*d*_*i*_, *t*_*j*_) should be ranked before (*d*_*i*_, *t*_*k*_). Then, *H* is divided into two parts, the training set and test set. Next, based on the training set, BPR is used to train the latent factor matrix of drugs and targets (nontargets). *F*^*D*^ represents the latent factor matrix of the drug (*F*^*D*^ ∈ ℝ^*m*×*f*^, *f* is the size of latent factor), *F*^*T*^ represents target (nontarget) latent factor matrix (*F*^*T*^ ∈ ℝ^*n*×*f*^, *f* is the size of latent factor). Among them, *F*_*i*_^*D*^ ∈ ℝ^1×*f*^ represents the latent factor of drug *d*_*i*_, and *F*_*j*_^*T*^ ∈ ℝ^1×*f*^ represents the latent factor of target (nontarget) *t*_*j*_. *r*_*i*,*j*_ = *F*_*i*_^*D*^∙*F*_*j*_^*T*^ is the predicted score for ranking the interaction of *d*_*i*_ and *t*_*j*_.

In order to improve the training of latent factors, we use the dual similarity regularization method based on the similarity theory to increase the latent distance between latent factors to increase the gap between the scores of different drug-target pairs.

**Figure 1:**
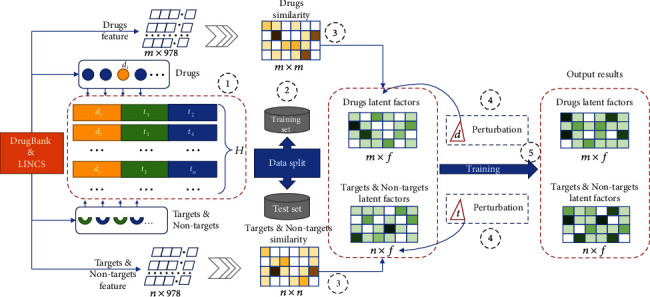
The flowchart of model training. (1) Generating ternary partial order set *H*. (2) Splitting *H* into a training set and a test set. (3) Calculating drug-drug and target-target similarity for improving latent factors. (4) Perturbation of latent factors for BPR. (5) Latent factor training.

Finally, gene expression data of LINCS project were treated as the features of drugs and targets to calculate drug-drug similarity and target-target similarity to improve training latent factors which represented key features of gene expression. Because the gene expression data are the observed values obtained from experiment, thus, the error between the observed value and the true value does exist. Therefore, latent factors of the drug and target (i.e., the model parameters) learned in this study can fluctuate within a certain range but the model's prediction results should be stable. Consequently, the perturbation factor Δ is introduced into the training process of *F*^*D*^ and *F*^*T*^ to make the trained model more robust. The overall process of model training is shown in Figure 1.

**Figure 2:**
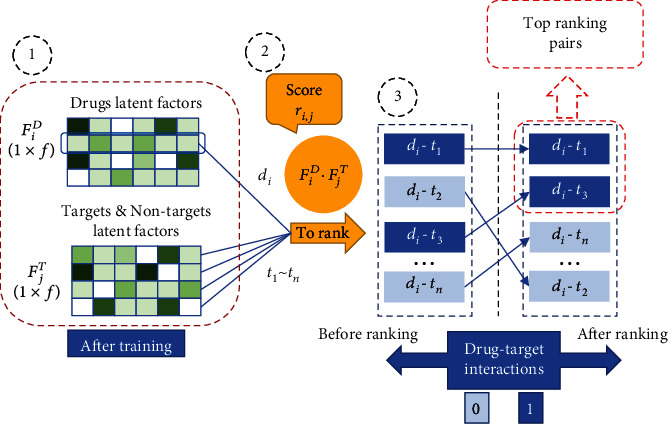
The flowchart of model prediction. (1) Latent factor matrix of *F*^*D*^ and *F*^*T*^ after training. (2) Calculating *r*_*i*,*j*_ for ranking. (3) Ranking drug-target pairs.

After the model is trained, calculate the value of *r*_*i*,*j*_ for all drug-target pairs, and sort them in a descending order. The top-ranked drug-target pairs are predicted as the interaction, and the bottom ranked drug-target pairs are predicted as the noninteraction. The prediction process is shown in Figure 2. Next, we will introduce the related methods in detail.

### 3.1. Bayesian Personalized Ranking

BPR is a pairwise LTR method. It learns in an implicit feedback manner through personalized ranking and is widely used in the recommendation systems [26].

As shown in Table 1, the numbers of drugs, targets, and interacting drug-target pairs in this study are all limited (for each cell line). Since one partially ordered triple was generated based on one positive sample and the corresponding negative sample, the number of partially ordered triples is also limited. Therefore, what we faced in this study were not only a small amount of partially ordered triples but also high-dimensional data. BPR is able to accurately predict even with a small amount of known data [26]. And BPR could map both drugs and targets into a shared low-dimensional latent feature space and to use this representation to calculate the probability of drug-target interactions to overcome the problem of high dimensionality [27].

According to the study of [26], BPR was derived for solving the personalized ranking task that only positive observations are available. In the problem of DTI prediction, only positive drug-target interactions can be directly obtained from the DrugBank database which is a key challenge in the DTI prediction problem. Hence, these advantages make BPR suitable for the DTI prediction problem.

In this study, we use this method to rank the score of drug-target pairs.

For *H*_*i*_ of *d*_*i*_(1 ≤ *i* ≤ *m*), we have

(1) $$\begin{array}{c} p\left(\theta \;|\;t_{j}{>}_{d_{i}}t_{k}\right)\propto p\left(t_{j}{>}_{d_{i}}t_{k}\;|\;\theta \right)p\left(\theta \right), \end{array}$$

where *θ* denotes the parameters of the model and *t*_*j*_>_*d*_*i*__*t*_*k*_ denotes that for *d*_*i*_ the possibility of interacting with *t*_*j*_ is greater than the possibility of interacting with *t*_*k*_. Since the interaction of *d*_*i*_ and *t*_*j*_ has no interference on the interaction of *d*_*i*_ and *t*_*k*_, all drug-target interactions are independent. The likelihood estimates for parameter *θ* are

(2) $$\begin{array}{c} \underset{\left(d_{i},t_{j},t_{k}\right)\in H_{i}}{\prod }p\left(t_{j}{>}_{d_{i}}t_{k}\;|\;\theta \right). \end{array}$$

In order to calculate *p*(*t*_*j*_>_*d*_*i*__*t*_*k*_ | *θ*), we use the logistic sigmoid function [26]:

(3) $$\begin{array}{c} p\left(t_{j}{>}_{d_{i}}t_{k}\;|\;\theta \right)=\sigma \left(r_{i,j}-r_{i,k}\right), \end{array}$$

where *σ*(∙) is the logistic sigmoid function and *σ*(*x*) = 1/(1 + *e*^−*x*^).

(*r*_*i*,*j*_ − *r*_*i*,*k*_) captures the ranking relation between *t*_*j*_ and *t*_*k*_ with the given *d*_*i*_. If *t*_*j*_ is more likely to interact with *d*_*i*_ than *t*_*k*_, then *r*_*i*,*j*_ ≥ *r*_*i*,*k*_ and (*r*_*i*,*j*_ − *r*_*i*,*k*_) ≥ 0. Otherwise, (*r*_*i*,*j*_ − *r*_*i*,*k*_) ≤ 0. Any standard collaborative filtering model can be applied to predict the value of (*r*_*i*,*j*_ − *r*_*i*,*k*_). Matrix factorization has been successfully applied in many studies [35–37]. Thus, the matrix factorization model is used in this study.

Next, consider *p*(*θ*) of formula (1). It is a Gaussian distribution with zero mean and variance-covariance matrix *λ*_*θ*_*I* [26], where *λ*_*θ*_ is a model-specific regularization parameter and *I* is an identity matrix, so

(4) $$\begin{array}{c} p\left(\theta \right)\sim N\left(0,\lambda_{\theta }\text{I}\right). \end{array}$$

According to formulas (2)–(4), the maximum posterior probability of the BPR method can now be rewritten as

(5) $$\begin{array}{c} \underset{\theta }{\max}\;L=\ln p\left(\theta \;|\;t_{j}{>}_{d_{i}}t_{k}\right)=\ln p\left(t_{j}{>}_{d_{i}}t_{k}\;|\;\theta \right)p\left(\theta \right)=\underset{\left(d_{i},t_{j},t_{k}\right)\in H_{i}}{\sum }\ln p\left(t_{j}{>}_{d_{i}}t_{k}\;|\;\theta \right)-\lambda_{\theta }{\left\|\theta \right\|}^{2}=\underset{\left(d_{i},t_{j},t_{k}\right)\in H_{i}}{\sum }\ln\sigma \left(r_{i,j}-r_{i,k}\right)-\lambda_{\theta }\left({\left\|F^{D}\right\|}^{2}+{\left\|F^{T}\right\|}^{2}\right), \end{array}$$

where ‖∙‖^2^ is an L2 regularization term.

From the maximum likelihood estimation for parameter *θ* in formula (5), an equivalent optimization objective formula can be obtained:

(6) $$\begin{array}{c} \underset{\theta }{\min}\;L_{\text{BPR}}\left(H_{i}\;|\;\theta \right)=\underset{\left(d,t_{i},t_{j}\right)\in H_{i}}{\sum }-\ln p\left(t_{j}{>}_{d_{i}}t_{k}\;|\;\theta \right)+\lambda_{\theta }{\left\|\theta \right\|}^{2}=\underset{\left(d_{i},t_{j},t_{k}\right)\in H_{i}}{\sum }-\ln\sigma \left(r_{i,j}-r_{i,k}\right)+\lambda_{\theta }\left({\left\|F^{D}\right\|}^{2}+{\left\|F^{T}\right\|}^{2}\right). \end{array}$$

### 3.2. Adversarial Bayesian Personalized Ranking

As mentioned, since the error between the observed value and the true value does exist, in order to enhance the robustness of the model, it is necessary to consider gene perturbations. It is unreasonable to add noise (such as changing the labels of training data) at the input layer. For example, modifying the training data (*d*_*i*_, *t*_*j*_, *t*_*k*_) to (*d*_*i*_, *t*_*k*_, *t*_*j*_) means that the noninteracting drug-target pair (*d*_*i*_, *t*_*k*_) is ranked higher than interacting drug-target pair (*d*_*i*_, *t*_*j*_). Obviously, the latent factors obtained by such training data are unreasonable. Therefore, it is necessary to add perturbations to the latent factors. For drug and target gene perturbations, we defined it as the perturbation factor that are added to Bayesian Personalized Ranking:

(7) $$\begin{array}{c} \underset{\Delta ,{\left\|\Delta \right\|}^{2}\leq \varepsilon }{\max}L_{\text{BPR}}\left(H_{i}\;|\;\theta +\Delta \right), \end{array}$$

where Δ is the gene perturbations on model parameters, *ε* controls the magnitude of adversarial perturbations, ‖∙‖^2^ denotes the L2 norm, and *θ* denotes the current model parameters (i.e., latent factors).

Δ can be optimal by adversarial perturbations Δ_*adv*_ as follows [28]:

(8) $$\begin{array}{c} \Delta_{\text{adv}}=\varepsilon \frac{\Gamma }{{\left\|\Gamma \right\|}^{2}},\Gamma =\frac{\partial L_{\text{BPR}}\left(H_{i}\;|\;\theta +\Delta \right)}{\partial \Delta }. \end{array}$$

Finally, we define the objective function of ABPR as follows:

(9) $$\begin{array}{c} L_{\text{AdvB}-\text{DTI}}\left(H_{i}\;|\;\theta \right)=L_{\text{BPR}}\left(H_{i}\;|\;\theta \right)+\lambda \Delta_{\text{adv}}, \end{array}$$

where *λ* controls the adversarial strength. The training process of AdvB-DTI can be expressed as playing a minimax game:

(10) $$\begin{array}{c} \underset{\theta }{\min}\underset{\Delta ,{\left\|\Delta \right\|}^{2}\leq \varepsilon }{\max}L_{\text{BPR}}\left(H_{i}\;|\;\theta \right)+\lambda L_{\text{BPR}}\left(H_{i}\;|\;\theta +\Delta \right), \end{array}$$

where the learning algorithm for model parameter latent factor *θ* is the minimizing player, which is aimed at obtaining accuracy prediction results. And the perturbation factor Δ acts as the maximizing player, which is aimed at identifying the worst-case perturbations against the current model. Finally, by playing this minimax game, it is able to make the model robust and simulate the error.

### 3.3. Dual Similarity Regularization

In the process of latent factors training, when drugs or targets are similar, their latent distance should be small. Conversely, when drugs or targets are different, their latent distance should be large. In order to meet this requirement, dual similarity regularization was introduced into this process.

In order to effectively combine the features of drugs and targets with matrix factorization methods, a Gaussian function needs to be introduced. Through this function, the features of drugs and targets can effectively influence the training of latent factors. Zheng et al. made the point that this function is sensitive to the latent distance of similarity between different drugs or targets [38]. The similarity between drugs (or targets) is negatively related to their latent distance. The function is defined as

(11) $$\begin{array}{c} \text{SimGaus}\left(S^{D},F^{D},d_{i}\right)=\overset{m}{\underset{j=1}{\sum }}{\left[S^{D}\left(i,j\right)-e^{-{\left\|F_{i}^{D}-F_{j}^{D}\right\|}^{2}}\right]}^{2},\\ S^{D}\left(i,j\right)=S^{D}\left(j,i\right)=\text{Sim}\left(d_{i},d_{j}\right). \end{array}$$

where *S*^*D*^ denotes drug-drug similarity matrix (*S*^*D*^ ∈ ℝ^*m*×*m*^), ‖∙‖^2^ denotes latent distance, and Sim(∙) is a similarity calculation method.

Similarly, we can obtain

(12) $$\begin{array}{c} \text{SimGaus}\left(S^{T},F^{T},t_{j}\right)=\overset{n}{\underset{k=1}{\sum }}{\left[S^{T}\left(j,k\right)-e^{-{\left\|F_{j}^{T}-F_{k}^{T}\right\|}^{2}}\right]}^{2},\\ S^{T}\left(j,k\right)=S^{T}\left(k,j\right)=\text{Sim}\left(t_{j},{\text{t}}_{k}\right), \end{array}$$

where *S*^*T*^ denotes target-target similarity matrix (*S*^*T*^ ∈ ℝ^*n*×*n*^).

Commonly used similarity calculation methods include cosine similarity, Tanimoto coefficient, structural similarity index, and Spearman's rank correlation coefficient.

Tanimoto coefficient is an extension of Intersection over Union. It can be used to measure the similarity of nonbinary features. It calculates the degree of correlation based on the magnitude of the feature vector. The closer the calculation result is to 1, the more similar the two vectors are. It is defined as

(13) $$\begin{array}{c} T\left(x,y\right)=\frac{xy}{{\left\|x\right\|}^{2}+{\left\|y\right\|}^{2}-xy}. \end{array}$$

Cosine similarity is determined by the angle between two vectors. The smaller the angle is, the more similar the two vectors are. It is defined as

(14) $$\begin{array}{c} \cos\left(x,y\right)=\frac{xy}{\left\|x\right\|\left\|y\right\|}. \end{array}$$

Structural similarity index is a common similarity calculation method used in computer vision to measure image quality [39]. It is defined as

(15) $$\begin{array}{c} \text{SSIM}\left(x,y\right)=\frac{\left(2\mu_{x}\mu_{y}+c_{1}\right)\left(2\sigma_{xy}+c_{2}\right)}{\left(\mu_{x}^{2}+\mu_{y}^{2}+c_{1}\right)\left(\sigma_{x}^{2}+\sigma_{y}^{2}+c_{2}\right)}, \end{array}$$

where *μ* is the mean, *σ*^2^ is the variance, *σ*_*xy*_ is the covariance, and *c*_1_ = 0.001 and *c*_2_ = 0.001 are constants to avoid the denominator being 0. The closer the calculation result is to 1, the more similar the two vectors are. Since technologies originating from computer vision have been widely used in DTI prediction in recent years, we attempt to use these methods to calculate the similarity between drugs and targets. Originally, *μ* is used as an estimate of the image brightness, *σ*^2^ is an estimate of the image contrast, and *σ*_*xy*_ is the measure of the similarity of the image structure. In our problem, *μ* is used as an estimate of the amount of change in gene expression, *σ*^2^ is used as an estimate of the relative change in gene expression, and *σ*_*xy*_ is used as an estimate of the change trend in gene expression.

Spearman's rank correlation coefficient is a similarity calculation method based on the ranking of feature data. It is defined as

(16) $$\begin{array}{c} \text{sprm}\left(x,y\right)=1-\frac{6\sum_{1}^{n}g_{i}^{2}}{n\left(n^{2}-1\right)}, \end{array}$$

where *g*_*i*_ is the difference in the ranks of *x*_*i*_ and *y*_*i*_ and the size of features is *n*. For example, if *x* = (1, 0, 3) and *y* = (1, 5, 2), then the rank of *x* = (2, 1, 3) and *y* = (1, 3, 2), thus *g* = (1, −2, 1). Similarly, the closer the similarity value is to 1, the more similar the two vectors are.

Because the Gaussian function is a numerically “sensitive” function, which means it can increase the impact of similarity on latent factor training. Thus, it can extend the latent distance between drugs (or targets) to increase the scores of different (*r*_*i*,*j*_ − *r*_*i*,*k*_), which is to increase the penalty for wrong rankings and optimize the training latent factors.

We use stochastic gradient descent to optimize the final objective formula:

(17) $$\begin{array}{c} \underset{\theta }{\min}\underset{\Delta ,{\left\|\Delta \right\|}^{2}\leq \varepsilon }{\max}\underset{H_{i}\subseteq H,\left(d_{i},t_{j},t_{k}\right)\in H_{i}}{\sum }L_{\text{BPR}}\left(H_{i}\;|\;\theta \right)+\lambda_{\text{adv}}{\text{L}}_{\text{BPR}}\left(H_{i}\;|\;\theta +\Delta \right)+\lambda_{\text{sim}}\left[\text{SimGaus}\left(S^{D},F^{D},d_{i}\right)+\text{SimGaus}\left(S^{T},F^{T},t_{j}\right)+\text{SimGaus}\left(S^{T},F^{T},t_{k}\right)\right], \end{array}$$

where *λ*_adv_ and *λ*_sim_ are adversarial and similar hyperparameters, respectively.

## 4. Experiment and Analysis

The experiments are designed to answer the following three questions:

- How do different similarity calculation methods affect the prediction results of the model?
- How do different numbers of latent factors, *λ*_sim_ and *λ*_adv_, impact the model's performance?
- Will our model (AdvB-DTI) outperform other prediction models?

### 4.1. Assessment Metrics

The assessment metrics used in the experiment are AUC [26], Top_*k* [40], and AUPR. AUC is defined as formula (18):

(18) $$\begin{array}{c} \text{AUC}=\frac{1}{\left|D\right|}\underset{d_{i}\epsilon D}{\sum }\frac{\;|\;\left\{\left(d_{i},t_{j},t_{k}\right)\;|\;r_{i,j}>r_{i,k},t_{j}\in T,t_{k}\in T,y_{i,j}=1,y_{i,k}=0\right\}\;|\;}{\left|H_{i}\right|}. \end{array}$$

The set of interacting drug-target pairs is called the positive set, and the set of noninteracting drug-target pairs is called the negative set. One drug-target pair is randomly selected from the positive set and the negative set, respectively. AUC means the probability that the model correctly predicts that the score of the drug-target pair from the positive set is larger than that of the drug-target pair from the negative set. AUC can better reflect the overall performance of the model. The larger the value of AUC is, the better the performance of the model is.

Top_*k*_*i*_ means for drug *d*_*i*_, among the *k* top-ranked drug-target pairs, the proportion of targets that interact with *d*_*i*_ in all the targets that interact with *d*_*i*_, which is defined as

(19) $$\begin{array}{c} Top\_k_{i}=\frac{\left|\left\{t_{j}\left\|\left\{t_{l}\;|\;r_{i,j}\leq r_{i,l},\forall t_{l}\in T,l\neq j\right\}\right.\;|\;\leq k-1,\forall t_{j}\in T,y_{i,j}=1\right\}\right|}{\left|\left\{t_{j}\;|\;\forall t_{j}\in T,y_{i,j}=1\right\}\right|}. \end{array}$$

Top_*k* is the average of all Top_*k*_*i*_ (1 ≤ *i* ≤ *m*). This assessment metric is equivalent to the recall rate. Top_*k* is defined as

(20) $$\begin{array}{c} \text{Top\_}k=\frac{1}{\left|D\right|}\underset{d_{i}\epsilon D}{\sum }\text{T}\text{op\_}k_{i}. \end{array}$$

The meaning of prec_*k*_*i*_ is, for drug *d*_*i*_, among the *k* top-ranked drug-target pairs, the proportion of targets that interact with *d*_*i*_. Its definition is shown in

(21) $$\begin{array}{c} prec\_k_{i}=\frac{\left|\left\{t_{j}\left\|\left\{t_{l}\;|\;r_{i,j}\leq r_{i,l},\forall t_{l}\in T,l\neq j\right\}\right.\;|\;\leq k-1,\forall t_{j}\in T,y_{i,j}=1\right\}\right|}{k}. \end{array}$$

prec_*k* is the average of all prec_*k*_*i*_(1 ≤ *i* ≤ *m*). This assessment metric is equivalent to the precision rate. prec_*k* is defined as

(22) $$\begin{array}{c} prec\_k=\frac{1}{\left|D\right|}\underset{d_{i}\epsilon D}{\sum }prec\_k_{i}. \end{array}$$

With different *k* values, drug *d*_*i*_ has different (Top_*k*_*i*_, prec_*k*_*i*_) pairs. Connecting all (Top_*k*_*i*_, prec_*k*_*i*_), we can obtain a curve. The area enclosed by the obtained curve and the coordinate axes is the AUPR_*i*_ of *d*_*i*_. AUPR_*i*_ is also a comprehensive assessment metric, which is defined as

(23) $$\begin{array}{c} {\text{AUPR}}_{i}={∯}_{\sigma \in \text{Top\_}k_{i}-\text{prec\_}k_{i}\text{ curve }}^{d_{i}}d\sigma . \end{array}$$

AUPR calculates the average of all AUPR_*i*_(1 ≤ *i* ≤ *m*). The closer the value is to 1, the better the model performance. It is defined as

(24) $$\begin{array}{c} \text{AUPR}=\frac{1}{\left|D\right|}\underset{d_{i}\epsilon D}{\sum }{\text{AUPR}}_{i} \end{array}$$

### 4.2. Results and Analysis

We adopted 5-fold nested cross-validation to evaluate the performance of the proposed method, which means that when analyzing the impact of hyperparameters, we only utilized the training set. For fair comparison, we tuned the parameters of each method so that they could achieve the best performance in comparison. The hyperparameters used in the experiments and their values are listed in Table 2.

Matrix factorization methods demonstrated their power and versatility in bioinformatics, for example, in the prediction of disease subtype alignment [41], drug repositioning [42], and protease target prediction [37]. Thus, we treat a state-of-the-art method which predicts DTI via DNN [8] as baseline and compare it with other state-of-the-art matrix factorization methods [28, 30].

#### 4.2.1. Comparative Experiment of Different Similarity Calculation Methods

Table 3 lists the results of comparative experiments of different similarity calculation methods performed independently in the seven cell lines. Four different methods were used for comparison.

From Table 3, it can be found that the prediction results of Tanimoto coefficient are better than those of the other three methods in seven cell lines. The performance based on Spearman's rank correlation coefficient is second to that of the Tanimoto coefficient in this experiment, and they are very close. The traditional cosine similarity calculation method was unstable in the experiment, and AUC is under 90% in cell lines A549 and HEPG2. The prediction performance of structural similarity index is similar to that of Spearman's rank correlation coefficient. Except cosine similarity, three similarity calculation methods all consider the value of the features in calculating the similarity. Cosine similarity only considers the angle between vectors. If two feature vectors have the same direction, they are considered similar regardless of value of the features. From the results of cosine similarity, it can be inferred that ignoring feature values may cause poor prediction performance. Therefore, based on the above results, Tanimoto coefficient is more suitable to the prediction problem.

#### 4.2.2. Impact of Different Settings of Hyperparameters

**Figure 3:**
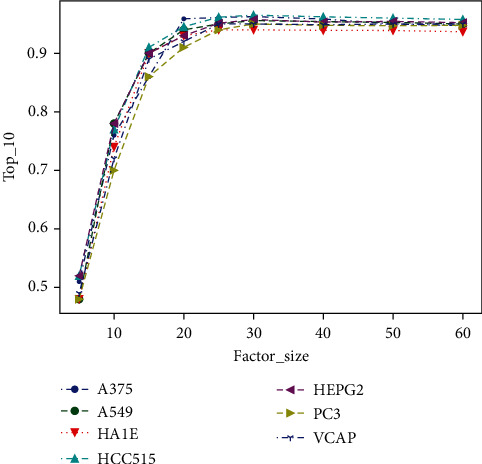
Impact of different numbers of latent factors on Top_10. Top_10 increases with factor_size and tends to be stable after factor_size is greater than 25.

Figure 3 reflects the relationship between the number of latent factors and the result of Top_10. For example, when factor_size = 5, Top_10 ≈ 0.5. It means that ten top-ranked drug-target pairs of a particular *d*_*i*_ predicted by the model contain about half of all interacting drug-target pairs of this drug (i.e., the recall rate is about 0.5). The meaning of latent factors is to map high-dimensional feature vectors to low-dimensional latent space and capture the implicit features of gene expression. The larger the size of the low-dimensional latent space, the more sufficient the feature information of the original high-dimensional drug and target expression can be that can be extracted. That is why the value of Top_10 significantly rises with the increase of the latent factor size. As shown in Figure 3, when the size of the latent factor increases to a critical size (e.g., factor_size > 25), the feature information is almost completely extracted, and the performance of AdvB-DTI becomes stable.

**Figure 4:**
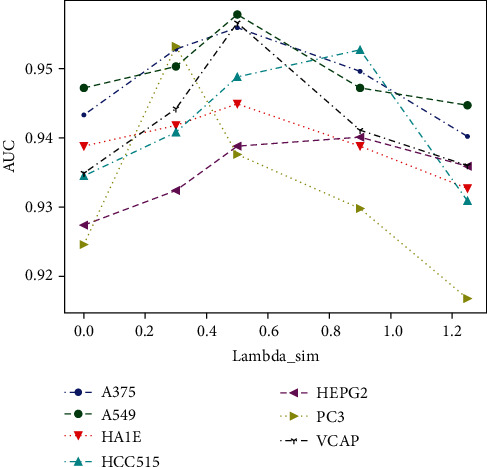
Impact of *λ*_sim_ on AUC. AUC increases with *λ*_sim_ but decreases when *λ*_sim_ is greater than a critical value.

Figure 4 shows the impact of *λ*_sim_ on the values of AUC. When dual similarity regularization was not used (i.e., *λ*_sim_ = 0), the values of AUC are lower than those using this method, which indicates that the method can improve the prediction performance.

Firstly, how does dual similarity regularization improve the training of latent factors? *r*_*i*,*j*_ is the score to rank. The ranking interval between different drug-target pairs is calculated by the difference of different scores. If *λ*_sim_ is set to a larger value, the latent distance between the drug and the target will also become large, and the same thing happens to different scores. Therefore, making the interval between different drug-target pairs increase will aggravate the penalty for the model when ranking errors occur during the training process. Thus, dual similarity regularization improves the training of latent factors.

Secondly, how to select a proper value for *λ*_sim_? The difference in *r*_*i*,*j*_ between different drug-target pairs increases with *λ*_sim_. Thus, the interval between different rankings increases. In cell lines with fewer positive samples, the model parameter *θ* will not be too large and increasing *λ*_sim_ can effectively improve the prediction performance. However, in cell lines with more positive samples, increasing *λ*_sim_ means that *θ* needs to increase beyond the limit of its regular term ‖*θ*‖^2^, so the model will be underfitting and the value of AUC decreases, as shown in Figure 4. AUC increases with *λ*_sim_ but decreases when *λ*_sim_ is greater than a critical value.

Therefore, in a cell line with fewer positive samples, a larger *λ*_sim_ will improve the prediction performance; however, in a cell line with more positive samples, a smaller *λ*_sim_ is suitable.

**Figure 5:**
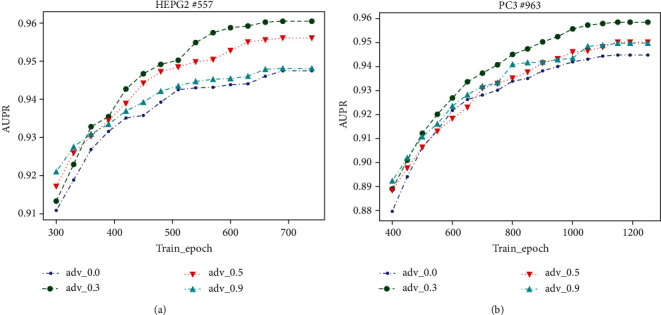
Impact of *λ*_adv_ on AUPR. For cell lines HEPG2 and PC3, the best performance of AUPR is achieved when *λ*_adv_ = 0.3.

In HEPG2 cell line, the number of positive samples is the smallest among the 7 cell lines. In PC3 cell lines, the number of positive samples is the largest among 7 cell lines. Therefore, in this experiment, we select these two cell lines as representatives to study the impact of *λ*_adv_ on prediction performance. In Figures 5(a) and 5(b), the curve of *λ*_adv_ = 0 represents that ABPR was not used in the model, and the other curves represent that ABPR was used in the model. In the early stages of training, the values of AUPR by using ABPR are better than those by not using ABPR. This is because when using ABPR, the parameters of the model could change within a certain range without changing the past prediction results, that is, learning new knowledge without forgetting the knowledge learned in the past. Thus, the prediction performance of the model can be effectively and quickly improved in the early stages of model training. Using ABPR as far as possible, the better performance will be obtained in the early stage of training.

Because of using Dual Similarity Regularization, the difference of scores of different drug-target pairs will increase; that is, the model parameters can withstand a certain range of perturbations to improve the model prediction performance. However, when the value of *λ*_adv_ exceeds a certain range, due to the constraints of the regular terms of the model parameters, they cannot resist excessive perturbations, which leads to the model being underfitted. Therefore, if *λ*_adv_ is given a large value, the model converges fast. The upper bound of model convergence depends on the ability of model parameters to resist the perturbations, which can be verified in the PC3 cell line. As shown in Figures 5(a) and 5(b), the larger *λ*_adv_ is, the lower the upper bound of model convergence. When *λ*_adv_ = 0.3, the model obtained the best prediction performance.

#### 4.2.3. Comparison with Other Methods

AdvB-DTI was compared with other state-of-the-art methods, and the prediction performances are listed in Table 4. The comparison methods include DNN [8], GMF [30], NeuMF [30], and AMF [28].

Xie et al. used a DNN framework [8] for DTI prediction based on transcriptome data in the L1000 database gathered from drug perturbation and gene knockout trials. We used the same configurations for DNN training.

NeuMF [30] is a deep learning matrix factorization framework for recommendation task with implicit feedback. In this method, DNN's input layer is defined as a latent vector instead of drug and target features. It is an improvement of GMF and DNN. To compare with NeuMF and GMF fairly, our model uses the same number of latent factors as NeuMF and GMF.

AMF [28] is a state-of-the-art approach designed for item recommendation with users' implicit feedback. It introduces the concept of ABPR and improves the method of BPR [26].

The results of DNN are used as baseline in Table 4. Since the DTI data are too sparse that each drug only has interactions with few targets, and DNN needs sufficient data for training, the performance of DNN is not attractive. DNN utilizes the transcriptome data as drug and target's feature. However, the transcriptome data has much noise, which also limits its performance. As shown in Table 4, other state-of-the-art matrix factorization methods' performances are better than that of the baseline.

When comparing AdvB-DTI with other state-of-the-art matrix factorization methods (NeuMF, GMF, and AMF), we could observe that only utilizing the relationship of drug and target could not guarantee an ideal prediction performance and efficiently exploiting the similarity of drug-drug and target-target will has a positive impact on the performance.

Notice that the performance of AMF is only second to that of AdvB-DTI. It demonstrates that adding perturbations to latent factors could make model learn noise, rather than utilize noise data to train model like DNN. That is the reason that AMF could achieve a better performance than other models except AdvB-DTI.

NDCG is mainly used for evaluating ranking methods [43]. As our model is a ranking method, we compared AdvB-DTI with AMF, which has the best performance in Table 4 except AdvB-DTI, as shown in Table 5. It can be seen from the results that AdvB-DTI outperforms AMF and it is verified that AdvB-DTI can effectively deal with the class imbalance problem and the problem of data sparsity.

Finally, we compared the computing resource consumption of these methods. All the algorithms were written using Python programming language and operated on a computer (Ubuntu 16.04.4 LTS, Core i9-7900X CPU, 3.3 GHz, 128 GB memory space). The algorithms were executed by CPU. We conducted 10 experiments in the cell line of A549, and each experiment concurrently executed 10 training procedures with 5-fold cross-validation. The average results are shown in Table 6.

It can be found that DNN has the largest memory cost because of its many parameters. GMF is a traditional matrix decomposition framework with simple structure and few parameters, so its memory cost is minimum. NeuMF is the framework of matrix decomposition combined with neural network, so its memory cost is slightly higher than that of GMF. AdvB-DTI improves AMF and NeuMF improves GMF. Comparing the two groups of models based on Tables 4 and 6, it can be found that the convergence time of the model is related to its final prediction performance, and the improvement of model performance may lead to the increase of training time. In addition, the neural network-based methods, such as DNN and NeuMF, take up a lot of CPU resources.

In summary, AdvB-DTI efficiently utilizes the similarity of drug-drug and target-target and the relationship of drugs and targets to train latent factors for drugs and targets to improve DTI prediction performance.

## 5. System Analysis of AdvB-DTI

After the comparison with other methods, we utilize top 1% of all the prediction results to demonstrate the strength of our method to predict novel DTIs. In order to verify our model, all the known DTIs which have been utilized in our model are removed for discussion in this section and the following analysis is in A375.

### 5.1. Examination of Results

To validate whether our prediction results are in accord with current knowledge, we examined the predicted DTIs using other DTI database, including TTD [44], IUPHARBPS [45], Matador [46], STITCH [47], DGIdb [48], and CTD [49].

**Figure 6:**
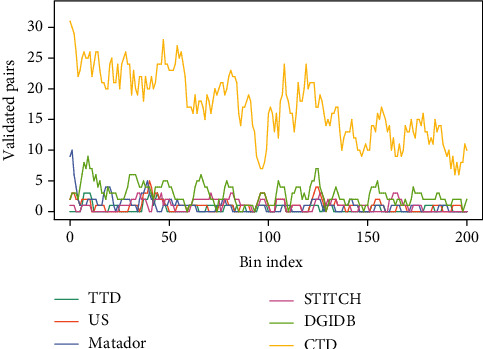
The overlap curves between predicted interactions and known DTIs.

We used *r*_*i*,*j*_ to rank all predicted DTIs and calculated pair counts that overlap between the predicted results and the interactions from other databases. Then, we counted the number of overlapping pairs in the sliding bins of 500 consecutive interactions (as shown in Figure 6). It suggests that our model can predict novel DTIs validated by known knowledge in other databases. Considering that DTIs in CTD database are curated from the published literature, these interactions are both direct (e.g., “chemical binds to protein”) and indirect (e.g., “chemical results in increased phosphorylation of a protein” via intermediate events); it is reasonable that CTD database covers a wider variety of drug-target interactions than other DTI databases.

### 5.2. Enrichment Analysis

In this study, the DrugBank database is considered the gold standard. The drug-target interactions from the DrugBank database are the most accurate and strict drug-target interactions. Besides the DrugBank database, there are some other databases containing a large amount of drug-target interaction data. These drug-target interaction data are much larger than the gold standard we used. Therefore, we compare our prediction results with the drug-target interactions contained in these databases. Here, the drug-target interactions in the IUPHARBPS database, STITCH database, CTD database, TTD database, Matador database, and DGIdb database were used. If our prediction results appear in other databases, it indicates that our prediction results are consistent with prior knowledge.

In order to characterize and quantify the appearance of predicted drug-target relationships (and known drug-target interactions) in other databases, we used the enrichment score and *P* value.

We calculated enrichment score (ES) as follows:

(25) $$\begin{array}{c} \text{ES}=\frac{kN}{nm}, \end{array}$$

where *k* is the number of predicted drug-target interactions that appear in the specified database (or the number of known drug-target interactions (i.e., drug-target interactions in our gold standard) that appear in the specified database); *N* is the number of all possible interactions between the drug set and the target set, that is, the drug-target interactions when the drug set and the target set are fully connected; *n* is the number of predicted drug-target interactions (or the number of known drug-target interactions in our gold standard); and *m* is the number of drug-target interactions in a specific database. And the interactions mentioned above only concern drugs and targets present in the gold standard.

Then, we used the hypergeometric distribution to calculate the *P* value as follows:

(26) $$\begin{array}{c} P\left(X\geq k\right)=\overset{\infty }{\underset{x=k}{\sum }}\frac{\left(m/x\right)\left(N-m/n-x\right)}{\left(N/n\right)}. \end{array}$$

FDR correction is used to correct the *P* values for multitesting [50].

As shown in Table 7, the known drug-target interactions and the drug-target interactions predicted using AdvB-DTI are significantly enriched on other datasets except for the STITCH database. Obviously, the known drug-target interactions (drug-target interactions in our gold standard) have larger enrichment scores and smaller *P* value than predicted drug-target interactions.

The results indicate that the drug-target interactions predicted by AdvB-DTI can be verified on other DTI datasets and have a potential practical value.

### 5.3. Drug Treatment Property

Drug ATC (Anatomical Therapeutic Chemical) label, which reflects drugs' therapeutic, pharmacological and chemical properties, is an important label of drugs. By comparing the distribution of drug ATC label in the known drug-target interactions and that of drug ATC label in the predicted drug-target interactions, we can find out which type of drug is more likely to be predicted to be associated with targets.

**Figure 7:**
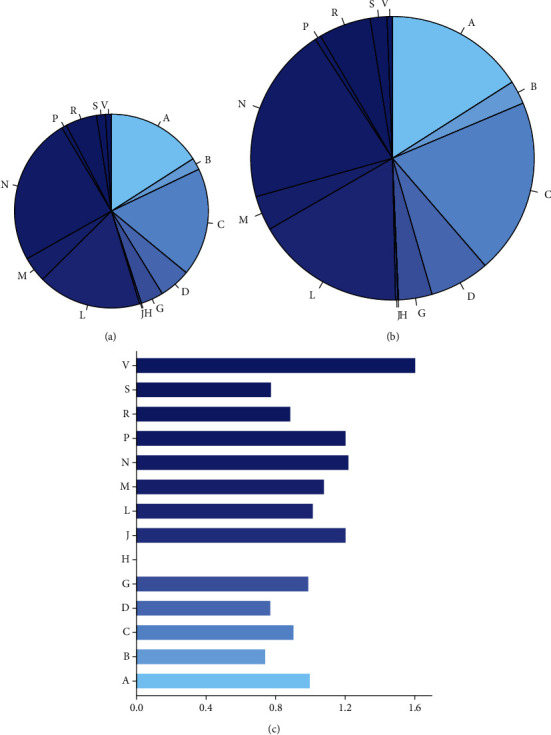
Distribution of ATC labels between DTIs in the known (a) and predicted (b) interactions. The relative ratio between known and predicted DTIs for each ATC label is shown in the right panel. ATC labels include the following: A—alimentary tract and metabolism; B—blood and blood-forming organs; C—cardiovascular system; D—dermatological; G—genitourinary system and sex hormones; H—systemic hormonal preparations, excluding sex hormones and insulins; J—anti-infectives for systemic use; L—antineoplastic and immunomodulating agents; M—musculoskeletal system; N—nervous system; P—antiparasitic products; R—respiratory system; S—sensory organs; and V—several others.

The distribution of drug ATC label in the known drug-target interactions and that of drug ATC label in the predicted drug-target interactions are illustrated in Figures 7(a) and 7(b). The relative ratio between known and predicted DTIs for each ATC label is shown in Figure 7(c). If there are 25% of drugs with ATC label A in the gold standard and 50% of drugs with ATC label A in the prediction result, the relative ratio is 0.25/0.5 = 0.5. The smaller the ratio, the more potential the drugs with that specific ATC label has to target proteins. So, the drugs with that specific ATC label should be studied further for broader use.

In Figure 7, the distributions of drug ATC labels for the gold standard and for the predictions (note that only the top 1% of all prediction results are taken) are almost the same. Notably, drugs with ATC label “B” (Blood and Blood Forming Organs) have a low relative ratio. In addition to A375, in most other cell lines, we also predicted more targets for drugs with ATC label “B”. The result suggests that drugs with ATC label “B” have more potential to target proteins and should be studied further for broader use.

## 6. Case Study

To illustrate the reliability of the prediction results of AdvB-DTI, we studied several cases in this section. These examples are all from our prediction results.

Olomoucine (CID: 4592) is a cyclin-dependent kinase inhibitor. For Olomoucine, its predicted target is MAPK3 through AdvB-DTI.

MAPK3 (Entrez ID: 5595) is a neighbor to the known target of Olomoucine (MAPK1, Entrez ID: 5594) in the protein-protein interaction (PPI) network. The PPI network, which contains 270,970 pairs of protein-protein interaction, is obtained from the BioGRID database [51]. By observing whether the edges (between two proteins) exist or not, we can judge whether drug known targets and predicted targets are neighbors in the PPI network. The closer two proteins are in the PPI network, the more likely they share the same functionality. Therefore, if the predicted targets are neighbors to the known targets of drugs, they might be targeted in the same way as known targets and the prediction results would be relatively reliable.

Indeed, recent research has shown that MAPK3 can be substantially inhibited by Olomoucine [52, 53]. This indicates that MAPK3 may be a novel target of Olomoucine.

Drug acetylsalicylic acid (commonly known or available as Aspirin, CID: 2244) is used for the treatment of pain and fever due to various causes. For acetylsalicylic acid, its predicted target is cyclin-dependent kinase-2 (CDK2) through AdvB-DTI.

CDK2 (Entrez ID: 1017) is a neighbor to two known targets of acetylsalicylic acid in the PPI network (Entrez IDs: 7157, 6256). Recent research has shown that CDK2 may be a novel target of acetylsalicylic acid [54]. This verifies our prediction.

CDK2 is a member of protein kinase family. It plays an important role in regulating various events of eukaryotic cell division cycle. Accumulated evidence indicated that overexpression of CDK2 should cause the abnormal regulation of cell-cycle, which would be directly associated with hyperproliferation in cancer cells [55]. Moreover, the examination of different kinds of human cancers, with defined molecular features, for their susceptibility to CDK2 inhibition has unveiled the scope in which CDK2 might represent a good therapeutic target [56–63].

Based on the above information, we speculate that acetylsalicylic acid, which is predicted to target CDK2, may have potential anticancer effects. Interestingly, the results of various studies have demonstrated that long-term use of acetylsalicylic acid may decrease the risk of various cancers, including colorectal, esophageal, breast, lung, prostate, liver, and skin cancer [64]. The predicted target CDK2 explains acetylsalicylic acid's anticancer effect to some extent.

Next example is the drug Panobinostat.

Panobinostat (CID: 6918837) is an oral deacetylase (DAC) inhibitor approved on February 23, 2015, by the FDA for the treatment of multiple myeloma. It acts as a nonselective histone deacetylase inhibitor (HDACi).

Histone deacetylase inhibitors (HDACis) are promising agents for cancer therapy. However, the mechanism(s) responsible for the efficacy of HDACi have not yet to be fully elucidated [65].

In this study, we predicted that Panobinostat's target is ATF3 through AdvB-DTI.

ATF3 (Entrez ID: 467) is a neighbor to six known targets of Panobinostat in the PPI network (Entrez IDs: 3065, 10013, 83933, 9759, 10014, 8841). As a proapoptotic factor, it plays a role in apoptosis and proliferation, two cellular processes critical for cancer progression [66–68]. And ATF3 has been postulated to be a tumor suppressor gene because it coordinates the expression of genes that may be linked to cancer [69].

Recent research has shown that ATF3 plays an important role in HDACi-induced apoptosis in multiple cell types [70]. HDACi can induce upregulation of ATF3 expression, thus eliciting the antitumor response [71].

Therefore, Panobinostat, as a HDACi, may treat myeloma by targeting ATF3.

Another interesting case is caffeine.

Caffeine (CID: 2519) is a widely consumed pharmacologically active product. It can be used for a variety of purposes, including the short-term treatment of apnea of prematurity in infants and pain relief and to avoid drowsiness [72].

For caffeine, its predicted targets include PTGS2 (Entrez ID: 5743) and PPARG (Entrez ID: 5468) through AdvB-DTI.

PTGS2 is one of two cyclooxygenases in humans. As a proinflammatory gene, it plays an important role in inflammation. Recent research has shown that caffeine treatment can reduce the expression of proinflammatory genes, including PTGS2 [73]. And caffeine can bind to PTGS2 acetaminophen complex with high energy, therefore modulating PTGS2 inhibition [74]. Furthermore, upregulation of PTGS2 is a critical oncogenic pathway in skin tumorigenesis. Han et al. verified that caffeine could block UVB-induced PTGS2 upregulation [75]. All these studies show that PTGS2 is a potential target for caffeine.

PPARG, another predicted target, is a ligand-activated transcription factor and important modulator for inflammation and lymphocyte homeostasis. There is also a study showing that PPARG were suppressed even with a low caffeine dose [76]. This suggests that PPARG is also a potential target for caffeine.

The above cases illustrate that our prediction results have a potential practical value and can provide clues to the analysis of the mechanism of action of certain drugs.

## 7. Conclusion

In this paper, we propose a DTI prediction framework named AdvB-DTI. Based on Bayesian Personalized Ranking, it uses the method of matrix factorization to predict DTIs. In order to solve the problem of existing DTI prediction methods based on matrix factorization, the proposed method combines the features of drugs and targets with the matrix factorization method. The advantage of this method over other similar methods is that BPR is combined with the perturbation factor and dual similarity regularization to make the model more robust and the training results more accurate. Experimental results verify that AdvB-DTI efficiently utilizes the similarity of drug-drug and target-target and the relationship of drugs and targets to train latent factors for drugs and targets to improve DTI prediction performance.

This study has the following positive impacts on the biomedical research.

Firstly, by integrating transcriptome data from drugs and genes, our model provides a practically useful and efficient tool for DTI prediction. The results of our study demonstrate that our method could discover reliable DTIs, thereby reducing the size of the search space for wet experiments and improving the drug discovery process.

Secondly, effective DTI prediction is achieved based on the transcriptome data. Our model used drug perturbation and gene knockout transcriptome data from the L1000 database of the LINCS project. Because the cost of experiments in LINCS project is relatively low, our prediction based on LINCS data not only ensures high accuracy but also has low cost.

Thirdly, our effective predictions verify that there is indeed a correlation between drug perturbation and the drug's target gene knockout at the transcriptional level. This correlation not only provides a basis for high-precision drug-target predictions but also provides a transcriptional perspective for the interpretation of drug mode of action. The correlation can also provide clues for future drug discovery.

## Figures and Tables

**Table 1:** Data volume of seven cell lines.

Cell line	Drug	Target	Nontarget	Interacting drug-target pair	Noninteracting drug-target pair
A375	520	363	2,754	796	1,432,080
A549	525	366	2,648	805	1,390,200
HA1E	533	372	2,707	818	1,442,831
HCC515	471	334	2,516	689	1,185,036
HEPG2	370	356	2,520	557	932,400
PC3	643	378	2,866	963	1,842,838
VCAP	521	377	3,003	809	1,564,563

**Table 2:** The parameters and settings used in the experiments.

Hyperparameter	Setting
factor_size	[5, 10, 15, 20, 25, 30, 40, 50, 60]
*λ*_sim_	[0,0.3,0.5,0.9,1.25]
*λ*_adv_	[0,0.3,0.5,0.9]
*ε*	0.1
*λ*_*θ*_	0.1
learning rate	0.03

**Table 3:** The impact of different similarity calculation methods on prediction performance in seven cell lines.

Cell line		Tanimoto	cos	SSIM	sprm
A375	AUC	0.9202	0.9088	0.9037	0.9119
	AUPR	0.9437	0.9160	0.9389	0.9436
A549	AUC	0.9347	0.8944	0.9247	0.9192
	AUPR	0.9477	0.9109	0.9425	0.9367
HA1E	AUC	0.9249	0.9174	0.9082	0.9035
	AUPR	0.9450	0.9401	0.9380	0.9389
HCC515	AUC	0.9163	0.9018	0.9045	0.9045
	AUPR	0.9403	0.9332	0.9377	0.9305
HEPG2	AUC	0.9259	0.8828	0.9144	0.9124
	AUPR	0.9303	0.9161	0.9249	0.9279
PC3	AUC	0.9306	0.9090	0.9116	0.9228
	AUPR	0.9581	0.9471	0.9459	0.9536
VCAP	AUC	0.9466	0.9102	0.9349	0.9349
	AUPR	0.9645	0.9558	0.9453	0.9543

**Table 4:** Comparison between AdvB-DTI and other methods.

Cell line		DNN	GMF	NeuMF	AMF	AdvB-DTI
A375	AUC	0.8984	0.8733	0.9013	0.9253	0.9564
	AUPR	0.8673	0.8385	0.8805	0.9350	0.9635
A549	AUC	0.9134	0.8927	0.9071	0.9246	0.9554
	AUPR	0.8724	0.8495	0.8986	0.9319	0.9673
HA1E	AUC	0.8938	0.8874	0.9052	0.9074	0.9428
	AUPR	0.8518	0.8424	0.8837	0.9137	0.9602
HCC515	AUC	0.8735	0.8912	0.8899	0.9009	0.9571
	AUPR	0.8259	0.8429	0.8493	0.9177	0.9654
HEPG2	AUC	0.8901	0.8742	0.8835	0.8896	0.9464
	AUPR	0.8135	0.8135	0.8297	0.8951	0.9624
PC3	AUC	0.8957	0.8774	0.8725	0.9205	0.9560
	AUPR	0.8647	0.8631	0.8538	0.9309	0.9632
VCAP	AUC	0.8975	0.9033	0.8920	0.9095	0.9556
	AUPR	0.8426	0.8388	0.8749	0.9126	0.9622

**Table 5:** Comparison of AdvB-DTI and AMF based on NDCG in seven cell lines.

Cell line	AdvB-DTI	AMF
A375	0.9469	0.9149
A549	0.9413	0.9136
HA1E	0.9373	0.8813
HCC515	0.9455	0.8951
HEPG2	0.9566	0.8854
PC3	0.9517	0.9098
VCAP	0.9535	0.9041

**Table 6:** Resources consumed by AdvB-DTI and other methods in the cell line of A549.

Method	Time (m) ↓	Memory (MB) ↓	CPU (%) ↓
DNN	5	518	33.8
GMF	5	80	36.4
NeuMF	6	101	44.7
AMF	7	230	5.7
AdvB-DTI	12	180	5.3

**Table 7:** Enrichment of drug-target interactions on other datasets.

	ES	PES	EP-Value	PEP-Value
TTD	107.91	3.60	292.06	1.20
STITCH	12.32	0.52	16.72	0.04
DGIdb	70.37	2.43	∞	2.88
CTD	9.18	1.73	134.46	6.10
Matador	59.28	5.87	131.13	6.10
IUPHARBPS	99.74	3.84	856.72	2.33

ES: enrichment score of known drug-target interactions; PES: enrichment score of predicted drug-target interactions; EP-Value: enrichment *P* value (after -lg10) of known drug-target interactions; PEP-Value: enrichment *P* value (after -lg10) of predicted drug-target interactions.

## Data Availability

Previously reported LINCS L1000 gene expression signature data were used to support this study and are available at DOI 10.1093/nar/gku476. This prior study (and dataset) is cited at relevant places within the text as a reference [31]. And previously reported DrugBank DTI data were used to support this study and are available at DOI 10.1093/nar/gkx1037. This prior study (and dataset) is cited at relevant places within the text as a reference [34].
